# Systems Immunology Analyses Following Porcine Respiratory and Reproductive Syndrome Virus Infection and Vaccination

**DOI:** 10.3389/fimmu.2021.779747

**Published:** 2021-12-16

**Authors:** Loïc Vivien Bocard, Andrew Robert Kick, Corinne Hug, Heidi Erika Lisa Lischer, Tobias Käser, Artur Summerfield

**Affiliations:** ^1^ Institute of Virology and Immunology, Mittelhäusern, Switzerland; ^2^ Department of Population Health and Pathobiology, College of Veterinary Medicine, North Carolina State University, Raleigh, NC, United States; ^3^ Department of Chemistry & Life Science, United States Military Academy, West Point, NY, United States; ^4^ Interfaculty Bioinformatics Unit, University of Bern, Bern, Switzerland; ^5^ Swiss Institute of Bioinformatics (SIB), Lausanne, Switzerland; ^6^ Department of Infectious Diseases and Pathobiology, Vetsuisse Faculty, University of Bern, Bern, Switzerland

**Keywords:** PRRSV (porcine reproductive and respiratory syndrome virus), transcriptomics, systems immunology, T-cell responses, innate response

## Abstract

This study was initiated to better understand the nature of innate immune responses and the relatively weak and delayed immune response against porcine reproductive and respiratory syndrome virus (PRRSV). Following modified live virus (MLV) vaccination or infection with two PRRSV-2 strains, we analyzed the transcriptome of peripheral blood mononuclear cells collected before and at three and seven days after vaccination or infection. We used blood transcriptional modules (BTMs)-based gene set enrichment analyses. BTMs related to innate immune processes were upregulated by PRRSV-2 strains but downregulated by MLV. In contrast, BTMs related to adaptive immune responses, in particular T cells and cell cycle, were downregulated by PRRSV-2 but upregulated by MLV. In addition, we found differences between the PRRSV strains. Only the more virulent strain induced a strong platelet activation, dendritic cell activation, interferon type I and plasma cell responses. We also calculated the correlations of BTM with the neutralizing antibody and the T-cell responses. Early downregulation (day 0–3) of dendritic cell and B-cell BTM correlated to both CD4 and CD8 T-cell responses. Furthermore, a late (day 3–7) upregulation of interferon type I modules strongly correlated to helper and regulatory T-cell responses, while inflammatory BTM upregulation correlated more to CD8 T-cell responses. BTM related to T cells had positive correlations at three days but negative associations at seven days post-infection. Taken together, this work contributes to resolve the complexity of the innate and adaptive immune responses against PRRSV and indicates a fundamentally different immune response to the less immunogenic MLV compared to field strains which induced robust adaptive immune responses. The identified correlates of T-cell responses will facilitate a rational approach to improve the immunogenicity of MLV.

## Introduction

Porcine reproductive and respiratory syndrome virus (PRRSV) is a positive single-stranded RNA virus, which can be divided into the PRRSV-1 and PRRSV-2 species; both cause respiratory symptoms or reproductive disorders leading to high levels of animal suffering and economical losses in pig farming (1). This is also caused by its ability to pave the way for respiratory co-infections (2). PRRSV represents a macrophage-tropic virus with a high level of immunomodulatory potential as a means to escape both innate and adaptive immune responses (1). In fact, the ability of PRRSV to escape innate immune responses in its replicating cells representing primarily macrophages and monocyte-derived cells is well-known at the molecular level: it results in a potent inhibition of type I interferon and other innate immune responses (1, 3). Nevertheless, plasmacytoid dendritic cells can sense PRRSV and PRRSV-infected cells and contribute to innate sensing of the virus (4, 5). At the level of adaptive immune responses to PRRSV, pigs can mount a protective immune response, mainly against similar virus strains; but this virus is described to induce both delayed and weak neutralizing antibody and T-cell responses (1, 6). There are probably a multitude of factors that contribute to the observed slow development of adaptive immune responses; these include epitope shielding by heavy glycosylation of the viral surface proteins, a possible involvement of regulatory T cells, the observation that PRRSV targets the thymus, and last but not least its interaction with the monocyte-macrophage system (1, 7–9). Nevertheless, it is still an enigma to precisely define what makes PRRSV so difficult to be controlled by the immune system.

Modified live virus (MLV) vaccines have been and are intensively used to reduce clinical signs, economical losses, and virus transmission; yet, these vaccines are not fulfilling many criteria of a good vaccine: besides safety concerns, currently available PRRSV vaccines only induce weak and delayed adaptive immune responses, and the reasons for this remain obscure (10).

Therefore, the present study was initiated to understand the relationship between innate and adaptive immune responses induced by PRRSV infection and vaccination. To this end, we utilized a transcriptomic-based systems immunology analysis pipeline that we previously established to analyze vaccine responses in pigs (11). It has been demonstrated that transcriptomic data from peripheral blood leukocytes can be most informative if analyzed using blood transcriptional modules (BTMs). These BTMs have been established by Li et al. and represent gene sets created on the basis of a high level of interactions (12). These BTMs provide information on metabolic processes relevant for immune responses and/or these responses themselves: changes in immune cell population distribution (physical BTM), cellular processes such as cell cycle and transcription, leukocyte‐specific signaling pathways, leukocyte migration, activation of particular immune cell types such as dendritic cells (DC) and T cells, inflammation, coagulation, platelet activation, antiviral responses, antigen presentation, and immunoglobulin production. Importantly, correlation analyses demonstrated that changes in the expression of certain BTMs a few hours or days after vaccination can predict adaptive immune responses (13, 14). We therefore hypothesized that using a systems immunology approach composed of both perturbation and correlation analyses will further our understanding of PRRSV immunology.

For our analyses we utilized peripheral blood mononuclear cells (PBMC) and immunological data from a comprehensive study that analyzed in detail T-cell (15) and antibody responses (16) after MLV vaccination and infection with two recent PRRSV-2 isolates.

## Material and Methods

### Ethics Statement

All peripheral blood samples and data utilized in this study were from previously published and approved animal experiment (15, 16); these publications describe the ethics.

### PRRSV Infection and Vaccination of Pigs With Downstream Cell Preparation

For these analyses, we utilized PBMC from an animal experiment for which detailed immunological data are available for both the T-cell (15) and antibody responses (16). Details on the animal treatment, blood and PBMC isolation are described in (15, 16). Shortly, PBMCs were isolated by density centrifugation from blood of animals inoculated with one of four solutions (six animals per group): (i) MOCK for the negative control group, (ii) 2 ml intramuscular MLV (lineage 5 VR2332 strain) vaccination, (iii) low pathogen PRRSV-2 [LP, strain NC134, 10^6^ tissue culture infectious dose (TCID)_50_, intranasal], or (iv) high-pathogenic PRRSV-2 (HP, strain NC174, 10^6^ TCID_50_, intranasal). After cell isolation, PBMCs were stored in liquid nitrogen (15). For this study, PBMC sampled before virus inoculations (day 0) and 3 and 7 days post inoculation (dpi) were shipped overnight (World Courier, New Hyde Park, NY, USA) on dry ice from Raleigh, NC, USA, to Mittelhäusern, Switzerland. Upon receipt, cells were returned to liquid nitrogen until the PMBCs were thawed for BTM analysis.

### Bioinformatics

PBMCs were thawed, and RNA was extracted using Trizol; the RNA quality was controlled with a Fragment Analyzer. All samples were found to have good quality (RNQ > 8) and were sequenced using an Illumina^®^ NovaSeq6000 sequencer (Illumina, San Diego, CA, USA). The quality of the reads was assessed using FastQC v. 0.11.2 (http://www.bioinformatics.babraham.ac.uk/projects/fastqc/). The reads were mapped to the *Sus scrofa* reference genome (Sscrofa_11.1) with HISAT2 v. 2.1.0 (17). FeatureCounts from Subread v. 1.5.3 was employed to count the number of reads overlapping with each gene, as specified in the Ensembl annotation build 91. The RNAseq data are available in the European Nucleotide Archive (www.ebi.ac.uk/ena) under the accession number PRJEB47066.

The Bioconductor package DESeq2 v. 1.18.1 was used to test for differential gene expression between the different time points for each infection/vaccine separately (18). For Venn diagrams we used an online tool available at https://bioinformatics.psb.ugent.be/webtools/Venn. Our specific interest was to identify genes where the change between two time points was different in vaccinated animals compared to the MOCK controls. We calculated the negative natural logarithm of the P-values with genes upregulated (positive log2-fold change values) and the natural logarithm of the P-values with genes downregulated. This list was used to rank the genes for the interaction term for a “ranked gene set enrichment analysis” (GSEA) (19) using the BTM as defined by Li et al. (20) and modified for pigs as described (11). A list of all BTM genes and their affiliation to the BTMs is found in the supplementary material.

To analyze the correlation between the BTM and the adaptive immune responses reported by Kick et al. (15, 16), we used the Gene Set Variation Analysis (GSVA): it estimates the variation of pathway activity over a sample population in an unsupervised manner using the BTM as gene sets (21). The single enrichment scores (ES) were calculated using the GSVA package within the R environment. With the aim to evaluate the evolution of gene expression over time and consider the starting point of each gene set for individual pigs, we subtracted the day 0 values from the day 3 and day 7 values, to obtain the ES values for days 3 (3/0) and days 7 (7/0), respectively. Additionally, the values of day 3 were subtracted from the values of day 7 to obtain a value reflecting changes between days 3 and 7 (7/3). The T-cell data used in the present study were obtained from previously published work and represented data obtained following *in vitro* restimulation of PBMC with homologous PRRSV/MLV and using proliferation as readout (15). The datasets are expressed as percentages and were generated using multicolor flow cytometry; details on the methodology are described in (15). Before using this data, the MOCK values were subtracted from the stimulated groups. These values were used to compute the correlation between the ES and the T-cell response data for each animal until 56 dpi. For the antibody responses, we used the data from a flow cytometry–based neutralization assay that generates a percentage value for virus inhibition (16). The 21 dpi endpoint was chosen to avoid the following plateau phase; it also focuses our analysis on early neutralizing antibody levels. The R squared value was used to qualitatively evaluate the correlation; only correlations with a p-value below 0.05 were considered and shown in the figures. All analyses were performed using R [version 4.0.2 (2020-06-22)] in the R studio environment (Version 1.3.1056). Plots were made with R package ggplot2 version 3.2.1

## Results

### Overview of Transcriptomic Data

The transcriptomic data presented in this report originate from PBMCs of an experiment that was previously described (15). Weaned piglets were inoculated with a commercial MLV or infected with one of two PRRSV-2 strains isolated from North Carolina (USA)—a low-pathogenic (LP) 1-3-4 strain, or a “high-pathogenic” (HP) 1-7-4 strain. Only the infected pigs developed fever and disease symptoms typical for PRRSV infection without clear differences between LP and HP viruses (15).

The analyses of differentially expressed genes (DEG) showed group-dependent kinetics in gene regulation. For the HP and MLV groups, strong up- and downregulations were found between day 3 and 7 (D7/3), whereas for LP a major modulation was found earlier at D3/0 (**Supplementary Figure 1A**). The number**s** of up- or downregulated DEG**s** at D7/0 were highest for the HP (4,400), followed by the LP (4,086), the MLV (1,888), and the Mock (1,163). To get an overview of common DEG between the treatment groups, we generated Venn diagrams (**Supplementary Figure 1B**). The highest numbers of common DEG**s** were found for the D7/0 comparisons, where the majority of DEG**s** were shared between the field viruses, but also almost half of the MLV DEG**s** were shared with the field strains. Considering the high number of DEG**s** in the LP group at D3/0 which contrasted with the HP and MLV group that showed a higher perturbation at D7/3, we compared the LP D3/0 with the HP7/3 and MLV7/3 (**Supplementary Figure 1B**, right panel). The results showed a high level of common DEG, indicating that at least in part similar DEG**s** were perturbed at earlier time points in the LP group. PCA only revealed some degree of separation by the factor time for the LP and HP groups (comparing D0 with D3 or D7 on PC1 or PC2, respectively; **Supplementary Figure 2**).

### Robust Early Innate Immune Responses Following PRRSV Infection but Not MLV Vaccination

**Figure 1:**
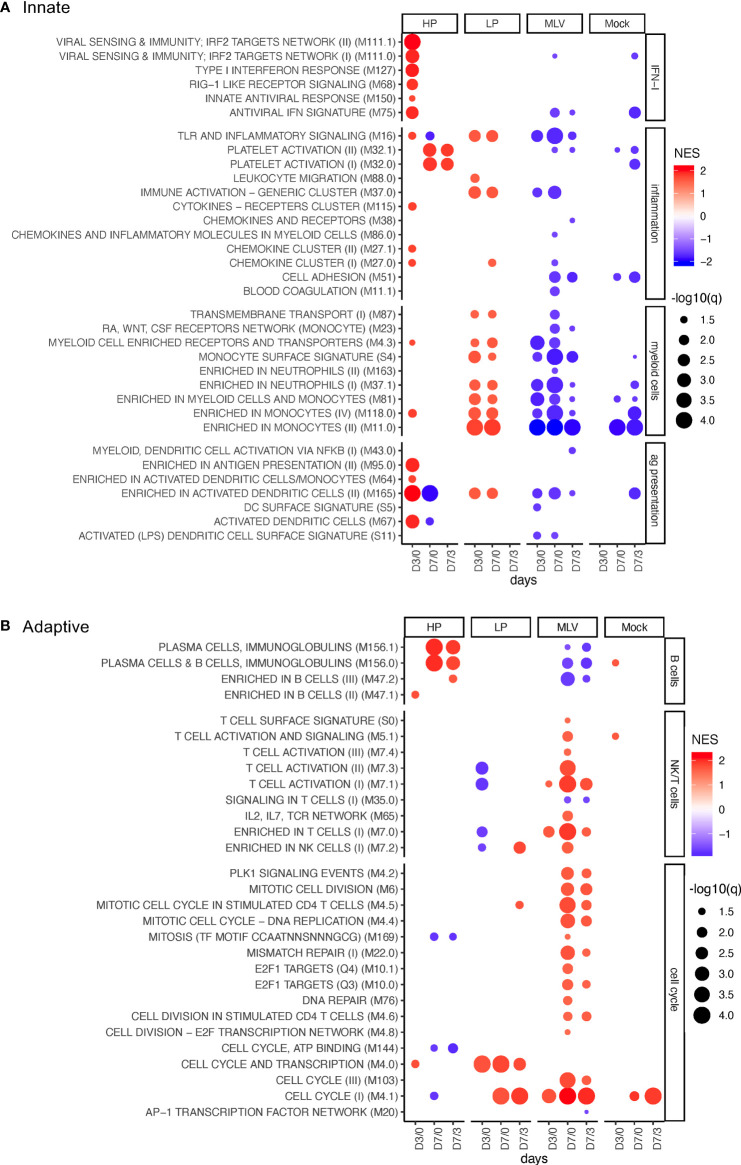
BTM perturbations induced by PRRSV and MLV. The bubble plots shows induction (red) or downregulation (blue) of BTM expression in PBMC following infection with HP, LP PRRSV2, MLV, or MOCK injection. The changes in BTM activity were determined for D0 to D3 (D3/0), from D0 to D7 (D7/0), and from D3 to D7 (D7/3). Treatments are defined in the x-axis. **(A, B)** show innate and adaptive BTM, respectively. On the left side of the heat map, the BTM families defined previously (11) are indicated. Circle sizes represent q-values, and color intensity represents the normalized enrichment scores (NES). A cutoff of an FDR of q<0.05 was employed.

We next analyzed the perturbations in gene expression by GSEA using the BTM as gene sets. **Figure 1A** shows BTMs more related to innate immune response; **Figure 1B** shows those more related to adaptive immune responses; and **Supplementary Figure 3** shows the perturbation of unclassified BTM (“various”).

#### IFN Type I Response Perturbation

Analyses of the BTM related to IFN type I responses revealed a major difference in the host response between the PRRSV-2 strains and the MLV: only the HP infection demonstrated a robust and early activation of antiviral BTM (**Figure 1A**).

#### Inflammatory Response BTM Perturbation

For inflammatory BTM, we found a very prominent induction of platelet activation BTM (M32) by HP only. On the other hand, the classical innate immune inflammatory BTM M16 and M37.0 were prominently induced by LP and not HP. Interestingly, these two and other inflammatory BTMs were downregulated by the MLV vaccine (**Figure 1A**).

#### Myeloid Cell BTM Perturbation

The transcriptomic perturbation analyses of myeloid BTM demonstrated an increase following infection with the LP strain only; in contrast, it was strongly decreased after MLV vaccination. The differently expressed BTMs were “enriched in monocytes” M11.0, M188.0, “monocyte surface signature” S4, and “enriched in myeloid cells and monocytes” M81, all indicating a relative increase of monocytes in this group. The changes were noted for all time comparisons including days 0 to 3 (D3/0) and D7/0 (**Figure 1A**).

#### Antigen Presentation and Dendritic Cell BTM Perturbation

For the BTM related to antigen presentation and DC, inoculation with HP led to a prominent upregulation of modules related to activated DC at early time points. For the LP, only M165 was induced. In contrast, MLV vaccination had no effect or induced a downregulation of BTM from this family (**Figure 1A**).

In summary, this data demonstrates innate responses were much more robust following PRRSV infection compared to the less immunogenic MLV. Furthermore, the type of innate BTM induced differed between the two field strains.

### Robust Plasma Cell Activation After HP but Not LP or MLV Inoculation

The modulated B-cell BTMs identified in this study were either physical BTM for B cells (M47) or modules mostly composed of immunoglobulin gene transcripts and plasma cell-specific transcripts (M156). Only, inoculation with HP induced these BTM at 7 dpi; in contrast, LP inoculation had no effects, and MLV vaccination reduced the expression of these B-cell BTM (**Figure 1B**).

### Robust Cell Cycle and T/NK-Cell BTM Activation With MLV Vaccination but Not Following LP or HP PRRSV-2 Infection

For BTM related to cell cycle, we observed a particularly strong increase in cell cycle BTM at D7/3 in the MLV group. M4.1 was also upregulated in both the MOCK and LP group. Interestingly, only in the LP group M4.0 was strongly induced for all three time point comparisons (**Figure 1B**).

Only the MLV vaccine induced physical BTM such as “enriched in T cells” M7.0, reflecting changes in the relative representation of T cells, as well as BTM indicative of T-cell activation and proliferation such as “T cell activation” (M7.1, M7.3) at D7/3. For the LP but not the HP strain, an early downregulation of some of these T-cells was found (**Figure 1B**).

Taken together, these perturbation analyses described in **Figure 1** demonstrate fundamental differences in the immune responses of the two PRRSV-2-infected groups, and even more when the field strains are compared to the MLV vaccinated animals; they also raise an important question: are these differences at least partly responsible for the higher and more rapid induction of adaptive immune responses in PRRSV-2 infected pigs? (15, 16). To address this question, the following chapter investigated the correlation between BTM modulation and the reported adaptive immune responses.

### BTM Correlates of Neutralizing Antibody Responses

To have sufficient replicates for the correlation analyses, we pooled the HP and LP animals as one group; yet, in **Figures 2**–**7** and **Supplementary Figures 4** and **5**, the MLV pigs were excluded based on their fundamentally different transcriptomic responses and the different route of inoculation. In contrast, **Supplementary Figures 6**–**12** show the results of a correlation analyses that included the MLV group.

**Figure 2:**
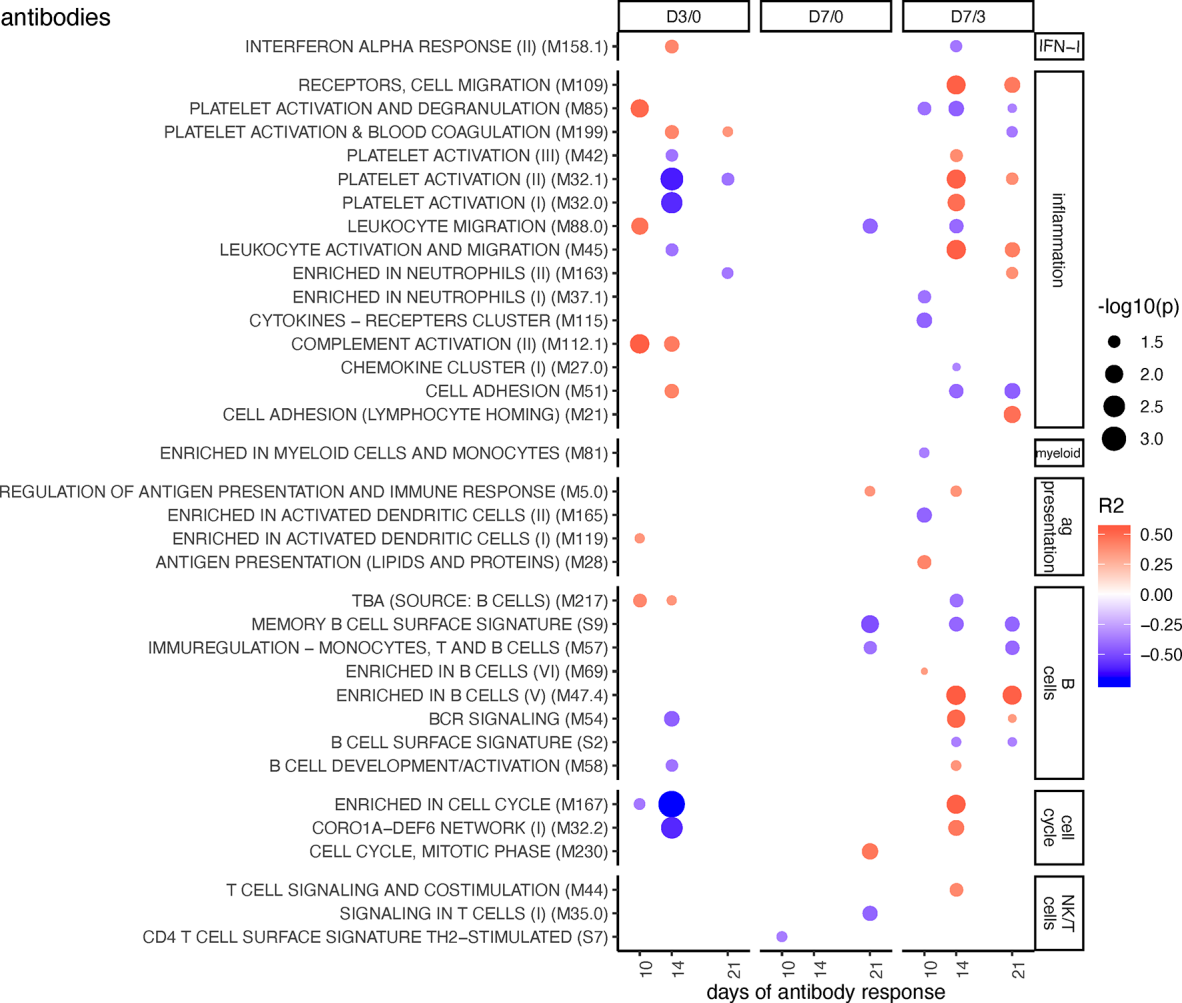
BTM correlating with anti-PRRSV inhibitory antibody levels. Time-dependent single sample enrichment scores for BTM were correlated with the AUC (0–21 dpi) of anti-PRRSV inhibitory antibodies [data from (16)]. R squared correlation coefficients for the BTM changes from D3/0, D7/0, and D7/3 are indicated by the color gradient (red for positive values, blue for negative values). Increased circle sizes indicate smaller p values (cutoff < 0.05). BTMs were grouped in families (11).

**Figure 2** shows BTM correlating with PRRSV inhibitory antibodies measured at day 10, 14, and 21. Considering that antibodies will accumulate over time, we focused on the BTM which were significantly correlating to the antibody levels at least at two time points. Early changes in BTM that correlated with the neutralizing antibody response included “blood coagulation” M199, “platelet activation” M32.1, “complement activation” M112.1, “enriched in cell cycle” M167, “enriched in plasma membrane” M124 (**Figure 2** and **Supplementary Figure 4**). At the later time points, when calculating changes between day 3 and 7 (D7/3), BTM related to cell migration (“receptors, cell migration” M109, and “leucocyte activation and migration” M45), to platelet activation (M32.1), and to B cells (“enriched in B cells” M47.4, “BCR signaling” M54) were found to positively correlate. At D7/3, there were many other correlating unclassified (various) BTM showing positive or negative correlations (**Supplementary Figure 4**).

Interestingly, these correlations were only partially reflecting the differences between MLV, and infection found in the perturbations analyses (**Figure 1**). This is noteworthy considering that the MLV induced a lower and delayed neutralizing antibody response when compared to the two field strains investigated here (16). Therefore, it was expected to see that if the MLV group was included into the analyses, a very different picture was obtained (**Supplementary Figure 6**). Now the D7/0 changes of many IFN-I, inflammation, myeloid cell, antigen presentation, and B cell BTM correlated positively, whereas most NK/T cell BTM correlated negatively with the antibody responses.

### BTM Correlations With PRRSV-Specific Proliferative T-Cell Responses

**Figure 3:**
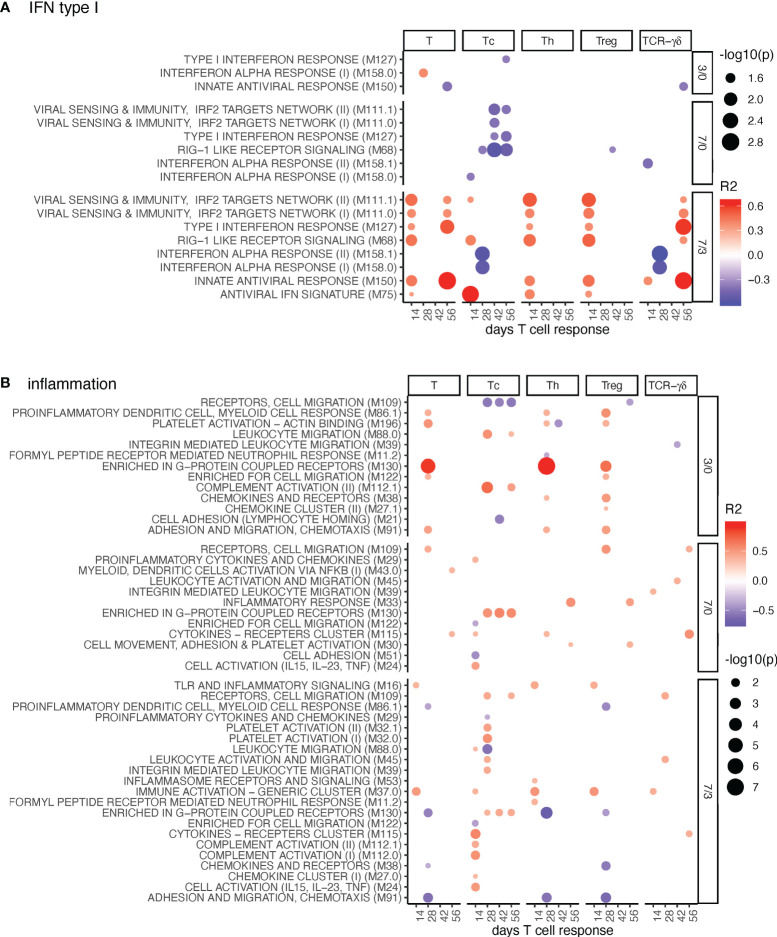
IFN-I and inflammation BTM correlating with T-cell priming. Time-dependent single-sample enrichment scores for BTM were correlated with the proliferative recall response of total CD3^+^ T cells, Tc, Th, and TCR-γδ T-cells measured at 14, 28, 42, and 56 days after LP or HP PRRSV-2 infection [x-axis, data from (15)]. R squared correlation coefficients for the BTM changes from D3/0, D7/0, and D7/3 are indicated by the color gradient (red for positive values, blue for negative values). Increased circle sizes indicate smaller p values (cutoff < 0.05). **(A)** shows IFN-I BTM and **(B)** inflammation BTM families (11).

**Figure 4:**
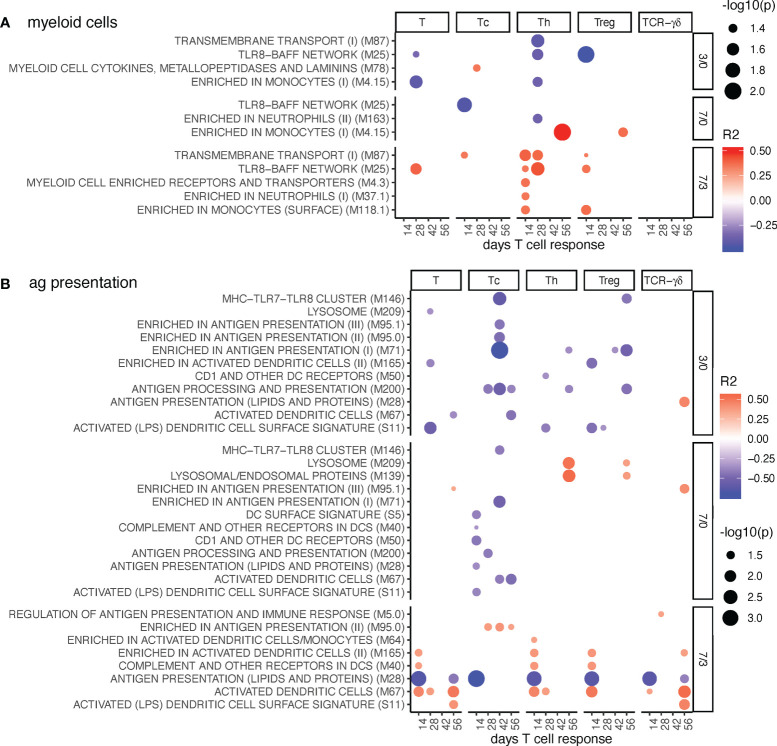
Myeloid cell and antigen presentation BTM correlating with T-cell priming. Time-dependent single-sample enrichment scores for BTM were correlated with the proliferative recall response of total CD3^+^ T cells, Tc, Th, and TCR-γδ T-cells measured at 14, 28, 42, and 56 days after LP or HP PRRSV-2 infection [x-axis, data from (15)]. R squared correlation coefficients for the BTM changes from D3/0, D7/0, and D7/3 are indicated by the color gradient (red for positive values, blue for negative values). Increased circle sizes indicate smaller p values (cutoff < 0.05). **(A)** shows myeloid cell BTM and **(B)** antigen presentation BTM families (11).

**Figure 5:**
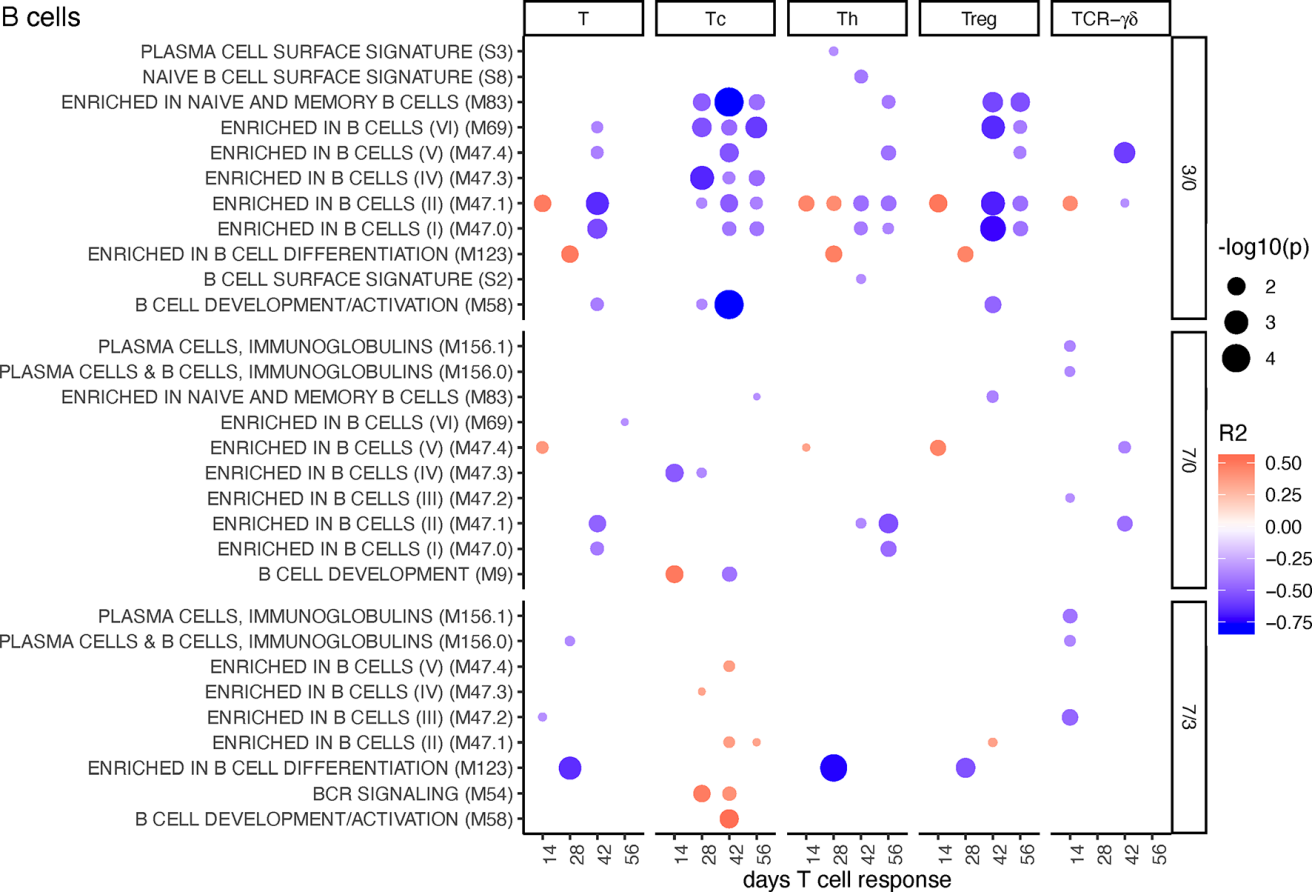
B-cell BTM correlating with T-cell priming. Time-dependent single-sample enrichment scores for BTM were correlated with the proliferative recall response of total CD3^+^ T cells, Tc, Th, and TCR-γδ T-cells measured at 14, 28, 42, and 56 days after LP or HP PRRSV-2 infection [x-axis, data from (15)]. R squared correlation coefficients for the BTM changes from D3/0, D7/0, and D7/3 are indicated by the color gradient (red for positive values, blue for negative values). Increased circle sizes indicate smaller p values (cutoff < 0.05).

**Figure 6:**
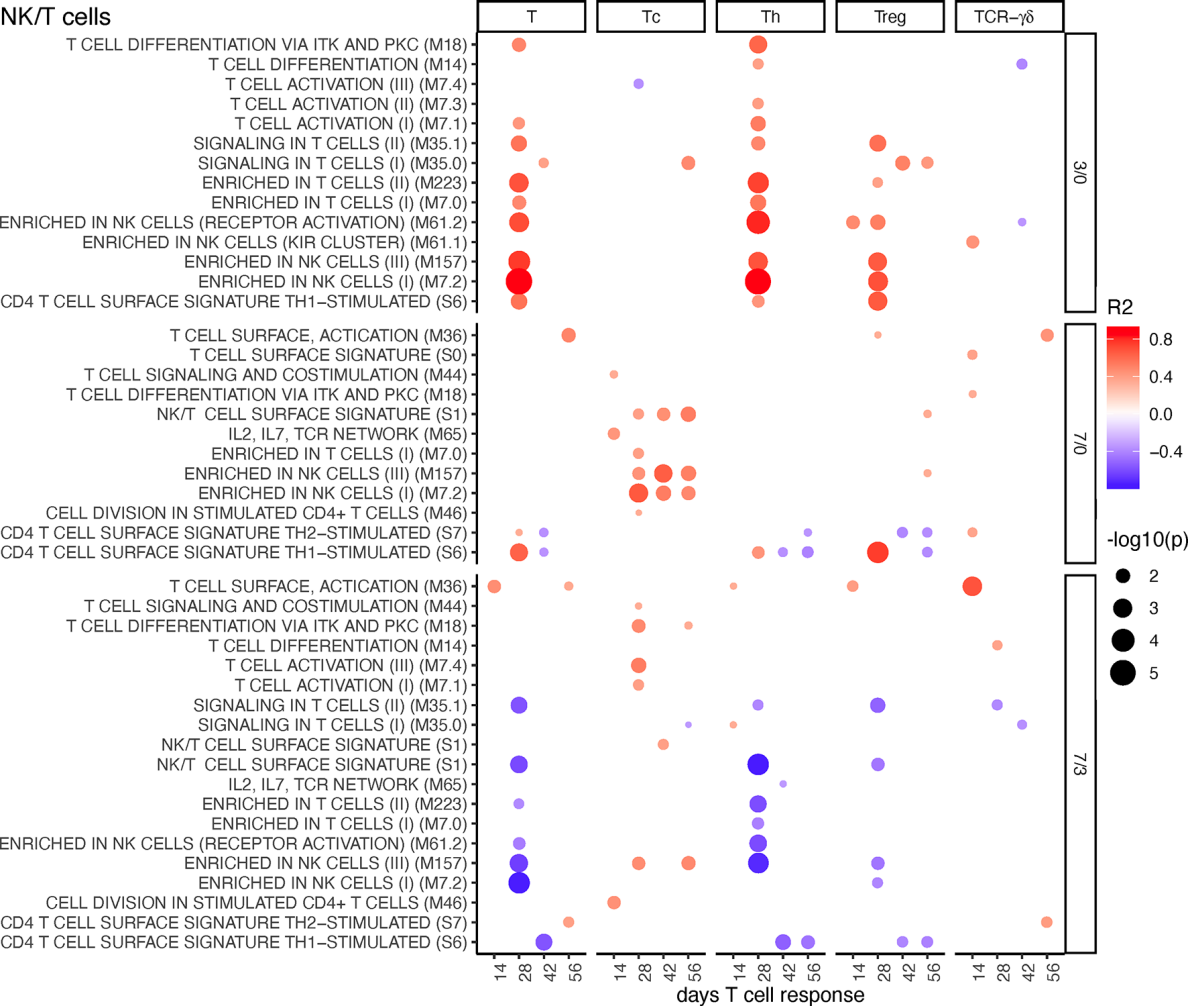
T/NK cell BTM correlating with T-cell priming. Time-dependent single-sample enrichment scores for BTM were correlated with the proliferative recall response of total CD3^+^ T cells, Tc, Th, and TCR-γδ T-cells measured at 14, 28, 42, and 56 days after LP or HP PRRSV-2 infection [x-axis, data from (15)]. R squared correlation coefficients for the BTM changes from D3/0, D7/0, and D7/3 are indicated by the color gradient (red for positive values, blue for negative values). Increased circle sizes indicate smaller p values (cutoff < 0.05).

As visible in **Figures 3**–**6**, we identified many BTM correlating with early T cell responses—at d14 and d28. For clarity of the text, we will mostly not mention the time points when these T-cell responses were measured. When referring in the text to early BTM changes, this represents the D3/0 comparison, while late BTM changes refers to D7/3 changes.

#### IFN Type I BTM and Inflammatory BTM

For the IFN type I BTM, we identified a prominent positive correlation between many BTM containing IFN response genes induced at late BTM time points (D7/3) with total T-cell, TCR γδ T-cells, Tc, Th, and Treg data (**Figure 3A**). For TCR-αβ T cells, these correlations were typically found at day 14, while for the γδ-T cells, the correlations were found at day 56 of T-cell analyses. It is important to note that the BTM “interferon alpha response” 158.0 and 158.1 are the only BTM containing IFN genes, whereas all others are mostly composed of IFN response genes. This could explain why these BTMs do not follow the same pattern and kinetics as the IFN response gene BTM.

For inflammatory BTM, the upregulation of “enriched in G-protein coupled receptors” M130 D3/0 stood out as a prominent early correlate for Th-cell responses (**Figure 3B**). Interestingly, for Tc responses, when compared to other T-cell subsets, a correlation with the induction of many more inflammatory BTM at D7/3 was evident (**Figure 3B**).

Inclusion of the MLV group in the correlation analyses confirmed many of the D7/3 correlations for the IFN-I BTM (**Supplementary Figure 7**). For the inflammation BTM, many additional positive correlations were found at all time points. Of note, we then found many D7/0 BTMs correlating with late Treg responses, indicating a possible link between early inflammation and late T-cell regulation (**Supplementary Figure 8**).

#### Myeloid Cells and Antigen Presentation BTM

Relatively few myeloid cell BTMs were found to correlate with Th and Treg proliferative responses (**Figure 4A**). The correlation was typically negative at early time point (D3/0) and positive at the late time point (D7/3).

Antigen presentation modules were generally negatively correlated at early but positively correlated at late BTM time points. This was seen for total T cells, T helper cells (Th), regulatory T cells (Treg), and cytotoxic T cells (Tc), but not for TCR-γδ T cells (**Figure 4B**). Interestingly, the early negative correlation of the antigen presentation BTM was particularly prominent for the Tc. The BTM “antigen presentation (lipids and proteins)” M28 stood out in showing a strong negative correlation at late time points to early T cell responses of all subsets. M28 contains only CD1 and MHC genes, while the positively correlating DC BTM M67, S11, M40, M165 are mainly composed of other genes related to innate activation of DC (12).

Inclusion of the MLV group into the correlation analyses resulted in a high number of positively correlating D7/0 myeloid cell and antigen presentation BTM. The correlations to the Th and Treg responses measured at day 56, but also for TCR-γδ T cells at day 42 were standing out (**Supplementary Figure 9**). Interestingly, M28 was still the only BTM with negative correlations at D7/3.

#### B-Cell BTM

For early changes in B cell BTM expression (D3/0), the correlation with Tc, Th, and Treg cell proliferative responses was mostly negative. This was particularly prominent for Tc and Treg (**Figure 5**). Late increase in some of these BTM (D7/3) such as several “enriched in B cells” BTM and “BCR signaling” had a positive correlation with Tc (**Figure 5**). Inclusion of the MLV group into the correlation analyses resulted in an overall similar profile of correlating BTM (**Supplementary Figure 10**).

#### NK/T Cells BTM

These BTMs had a positive correlation at the early time point (D3/0) and negative associations at the late time point (D7/3) to total T cells, Th, and Treg responses. Interestingly, the latter did not apply to Tc responses, which correlated positively to NK/T cell BTM upregulations at D7/0. It is noteworthy that these responses included both physical as well as activation modules. For the D7/3 T/NK cell BTM, several negative correlations to Th cells were found (**Figure 6**). Inclusion of the MLV group into the correlation analyses partially changed the profile of correlations. At D3/0, only the “signaling in T cells” BTM M35.0 and M35.1 now stood out as prominent positively correlating modules. At D7/0, the negative correlations were dominating (**Supplementary Figure 11**).

#### Cell Cycle BTM

**Figure 7:**
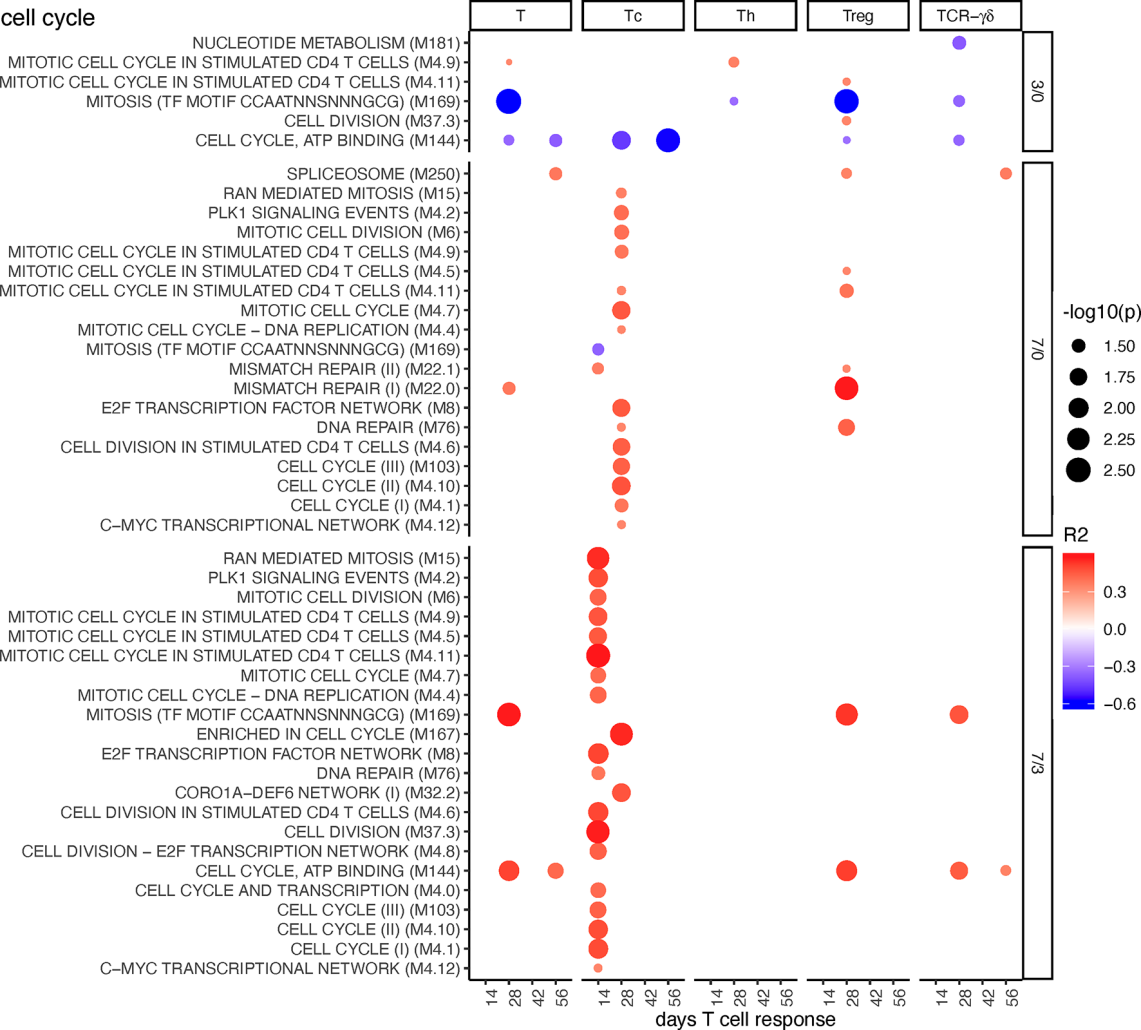
Cell cycle BTM correlating with T-cell priming. Time-dependent single-sample enrichment scores for BTM were correlated with the proliferative recall response of total CD3^+^ T cells, Tc, Th, and TCR-γδ T-cells measured at 14, 28, 42, and 56 days after LP or HP PRRSV-2 infection [x-axis, data from (15)]. R squared correlation coefficients for the BTM changes from D3/0, D7/0, and D7/3 are indicated by the color gradient (red for positive values, blue for negative values). Increased circle sizes indicate smaller p values (cutoff < 0.05).

A few negative correlations of cell cycle BTM to total T cells, Treg, and Tc responses were found at early time points (D3/0). In contrast, at late time points (D7/0 and D7/3), many BTMs correlated positively with Tc, in particular (**Figure 7**). Inclusion of the MLV group into the correlation analyses resulted in similar of correlations, but now included Tregs (**Supplementary Figure 12**).

## Discussion

The present study was initiated on the one side to better understand the early interactions of PRRSV-2 during infection and MLV vaccination with the porcine immune system and on the other side to identify immune response elements that may be relevant for protective immune responses, disease development, and vaccination. To this end we performed a combination of perturbation and correlation analyses using transcriptomic data from PBMC and employed published T-cell and antibody response data (15, 16).

A first finding of our work was that intramuscular MLV vaccination often caused an opposite regulation of BTM when compared to intranasal inoculation with virulent field viruses. In particular, we observed a prominent downregulation of inflammatory, myeloid cell, DC, and B cell BTM; in contrast, T/NK cell and cell cycle BTMs were prominently upregulated by the vaccine only. Although the difference in application route might explain parts of this opposing regulation, it should be noted that the kinetics and heights of viremia were similar between the groups (15). The differences in the transcriptome of MLV vaccinated and PRRSV-2 infected PBMCs are interesting in the light of their difference in inducing adaptive immune responses: compared to inoculation with either PRRSV-2 strain, MLV vaccination induced a weaker T-cell response and a delayed neutralizing antibody response (15, 16). The development of the innate BTM (**Figure 1A**) in the MLV group could reflect migration patterns of innate immune cells from the blood to the site of injection and/or lymphoid tissue. The increase in inflammatory, myeloid cell, and DC modules following infection possibly reflects more systemic and intensive innate immune responses that spill over into the blood. On the other hand, the prominent increase of T-cell activation and cell cycle modules at day 7 found after MLV could be caused by activated lymphocytes that rapidly recirculate. The lack of this response following infection seems surprising considering that it was the infected pigs that showed stronger and earlier adaptive immune responses. A possible explanation is that recirculating lymphocytes were strongly attracted to the sites of inflammation which were missing or scarce following MLV injection. A better understanding of how the peripheral blood can reflect events in lymphoid tissue and effector sites will require sampling of more time points and, if possible, also lymphoid and inflamed tissues.

When comparing the two PRRSV field strains, the DSeq2 and BTM-based analyses revealed clear differences between the strains. The LP had more prominent early perturbations of DEG at D3/0, whereas these perturbations were occurring mainly between D7/3 for the HP strain. In the BTM analyses, only the HP strain induced a strong IFN type I response, DC and platelet activation, as well as a plasma cell response; in contrast, the LP had a more prominent inflammatory and myeloid cell response. Possible explanations for these differences could be differences in the kinetics of replication and cellular tropism. Investigating such factors would require a systematic virological analysis of tissues, which was not performed in the present study. A possible working hypothesis for future investigations could therefore be that the balance between inflammatory and antiviral response would relate to differences in strain-dependent PRRSV virulence.

To better understand which of these BTMs are associated with adaptive immune responses, we performed correlation analyses focusing only on the field PRRSV infections. It was difficult to identify a clear pattern of correlations for the neutralizing antibody responses, although an early downregulation of a number of BTM from various families were associated with higher antibody levels. A possible reason for this was the relatively low level of variation in neutralizing antibody responses between the animals (16).

In contrast, a clear pattern of correlations was identified for the TCR-αβ T-cell responses. Typically, good TCR- αβ T-cell responses were associated with early downregulations of DC and B-cell BTM and late upregulation of inflammation BTM. For Th and Treg cells, a prominent association with late IFN type I responses was found. The correlation pattern for Tc proliferation was associated with a more prominent early downregulation of antigen presentation modules and more strongly associated with a late inflammatory BTM response. Also, the association to late NK/T cell BTM was different (negative for Th, positive for Tc). This was remarkable and indicated distinct immunological processes and different kinetics could be involved in CD4 and CD8 T cell priming and memory T-cell formation. When focusing on the association of BTM changes to TCR-γδ T cell responses, we found relatively few correlations. This reflects their different mode of activation compared to that of TCR-αβ T cells. Nevertheless, late TCR-γδ T cell responses (day 56) correlated strongly to some IFN response BTM measured D7/3.

An important observation for the design of future studies was also that by far most correlations were restricted to the T-cell response measured at d14 and d28 with the latter often representing the peak of the response (15). Our explanation for this is that T-cell recirculation and homing is impacting the later phases of the T-cell response measured in the blood and that these processes are less related to the early innate responses measured as BTM changes.

Altogether, some of the BTM, in particular those related with IFN type I responses, found to be associated with good T-cell responses were reminiscent of those described for yellow fever vaccine in humans (12) or for a dengue vaccine in non-human primates (22). Importantly, the BTM correlation patterns for inactivated vaccines differ from live virus vaccines. For inactivated/subunit vaccines, the early upregulation of myeloid cell and DC BTM represents an important correlate of adaptive immune responses in humans (12, 23), sheep (24), and pigs (11).

We also generated a set of correlation data calculated by including the MLV group. The interpretation of these data needs to take the different perturbations caused by the MLV inoculation as compared to the LP/HP infections, the different kinetics of adaptive immune responses, and the different route of inoculation into consideration. Nevertheless, many correlates of T-cell responses were confirmed, indicating that these could be broadly applicable correlates for PRRSV.

If and how the route of inoculation impacts protection and correlates of protection represents an important future research question, although it was recently shown that the MLV vaccine can also be protective when applied *via* the intranasal route (25). To address the limitations of the present work, we recommend that future studies are designed with increased number of animals per group to enable group-specific correlation analyses. These studies should extend the duration of sampling as the kinetics of immune responses are likely to differ between different viruses, vaccine strains, different doses, and routes of inoculation. Furthermore, the inclusion of a detailed virological analyses to understand the relationship between immune responses and virus replication kinetics and tissue tropism would be very valuable.

An important future aim will be identifying how such data can be employed to improve PRRSV MLV. One pathway could be to increase the ability of the vaccine to induce later IFN-I and certain inflammatory reactions with the aim to enhance the ability of vaccines to induce potent T-cell responses. In fact, such approaches have demonstrated promising results (26); however, too much interferon type I can also prevent vaccine immunogenicity by blocking its replication (27). Clearly, finding the balance between immunogenicity and attenuation is a major challenge; but we propose that the systematic employment of a systems immunology approach will pave the way to improved MLV vaccines.

## Data Availability Statement

The datasets presented in this study can be found in online repositories. The names of the repository/repositories and accession number(s) can be found below: https://www.ebi.ac.uk/ena, PRJEB47066.

## Author Contributions

Conceptualization and study design: AS, LB, AK, HL, and TK. Formal analysis, AS, CH, HL, and LB. Supervision: AS. Writing—original draft: AS. Writing—review and editing, all authors. Funding acquisition: TK and AS.

## Conflict of Interest

The authors declare that the research was conducted in the absence of any commercial or financial relationships that could be construed as a potential conflict of interest.

## Publisher’s Note

All claims expressed in this article are solely those of the authors and do not necessarily represent those of their affiliated organizations, or those of the publisher, the editors and the reviewers. Any product that may be evaluated in this article, or claim that may be made by its manufacturer, is not guaranteed or endorsed by the publisher.
