# Supplementary material for: Systems Immunology Analyses Following Porcine Respiratory and Reproductive Syndrome Virus Infection and Vaccination
Source: Front Immunol. 2021 Dec 16;12:779747. doi: 10.3389/fimmu.2021.779747 (PMC8716554; doi:10.3389/fimmu.2021.779747)
Supplement: Supplementary file 1 [file DataSheet_1.pdf]

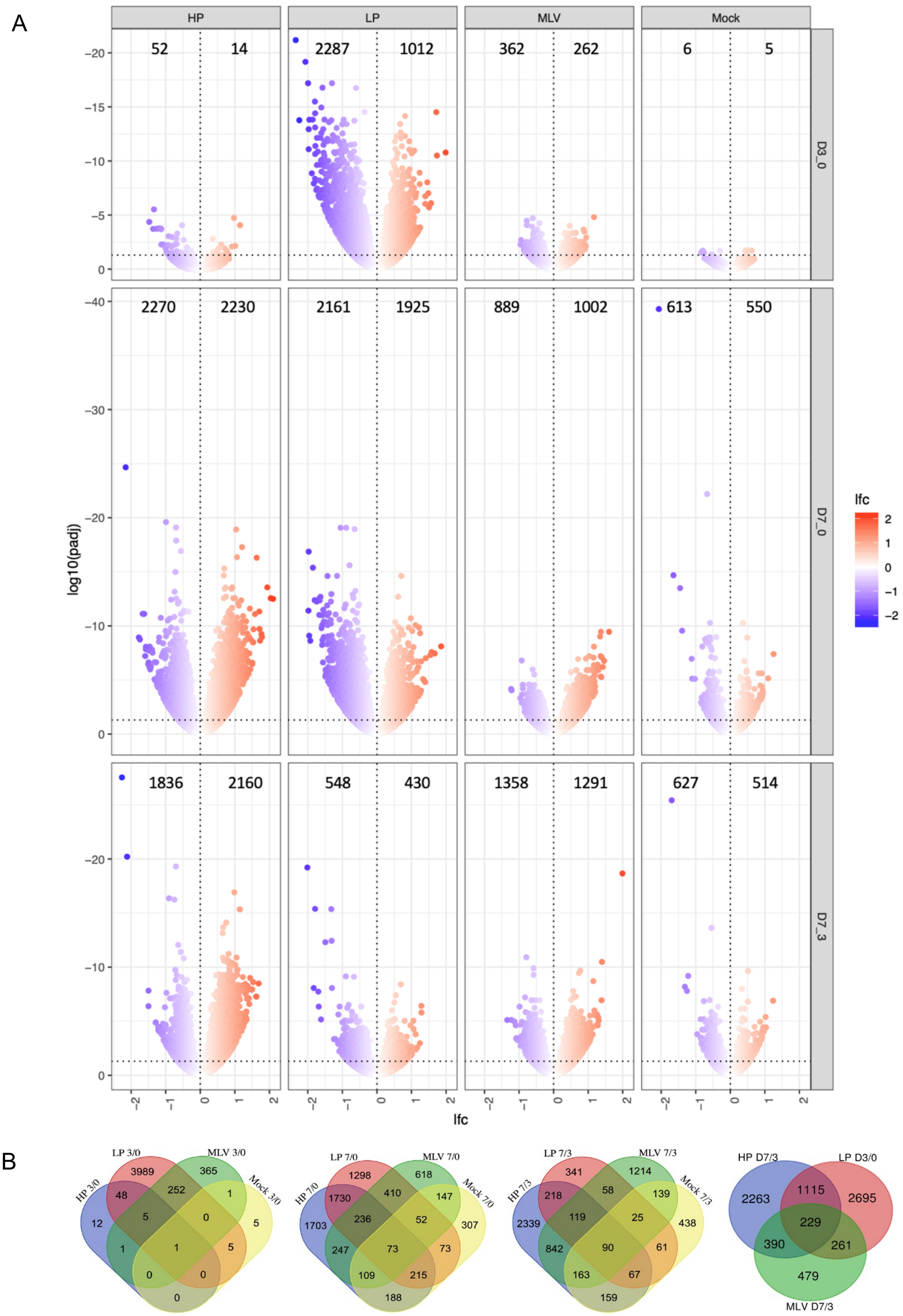

Supplementary Figure 1. Differentially expressed genes (DEG) for the three time point comparisons (D3/0, D7/0 and D7/3) in the four treatment groups (HP, LP, MLV, Mock). A. Volcano plot of up- and downregulated genes. The numbers of genes with a cut-off of padj<0.05 is shown. Red and blue color intensities represent of log2-fold-change (lfc). The vertical dotted line is placed at this cut-off. B. Venn diagrams for DEG (cut-off at padj 0.05) for the D3/0, the D7/0 and the D7/3 comparison (left to right). The left plot compared the DEG of LP at D3/0 with HP at D7/3 and MLV at D7/3.

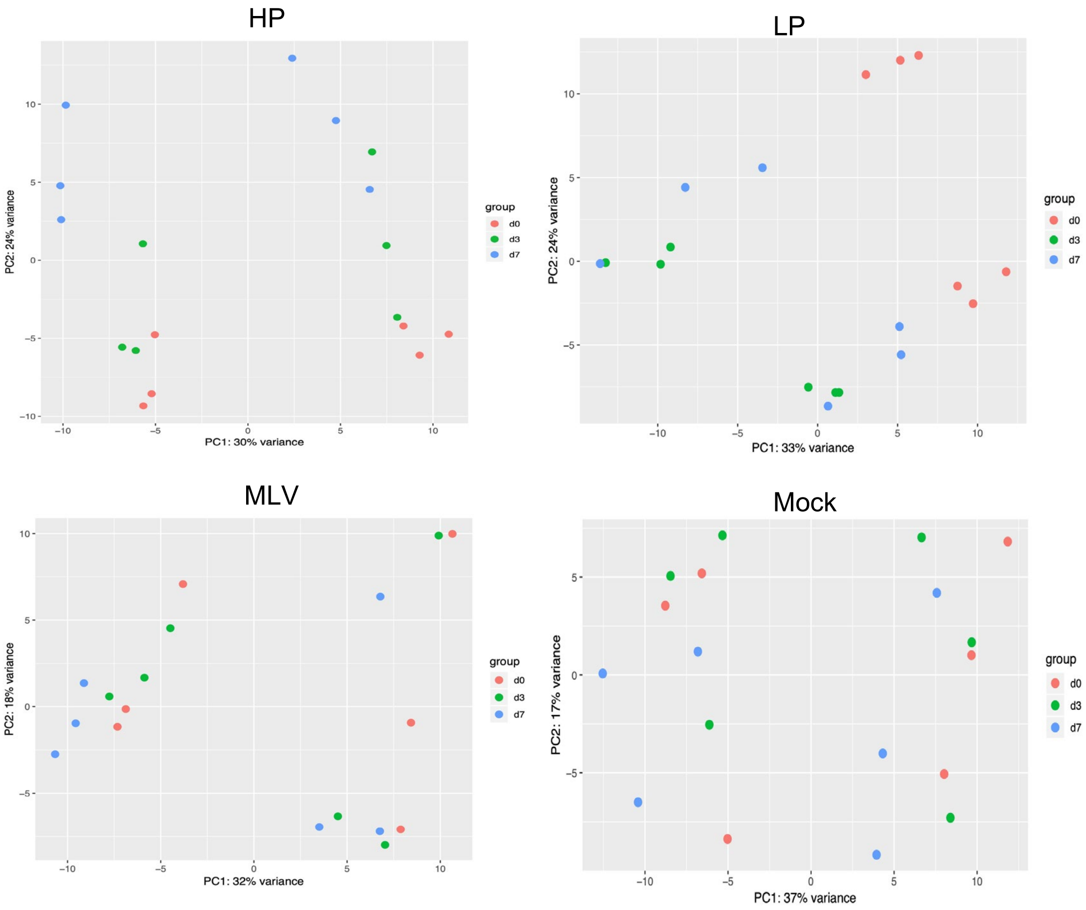

Supplementary Figure 2. Principle component analyses based on the 500 most variable genes analyzed group by group.

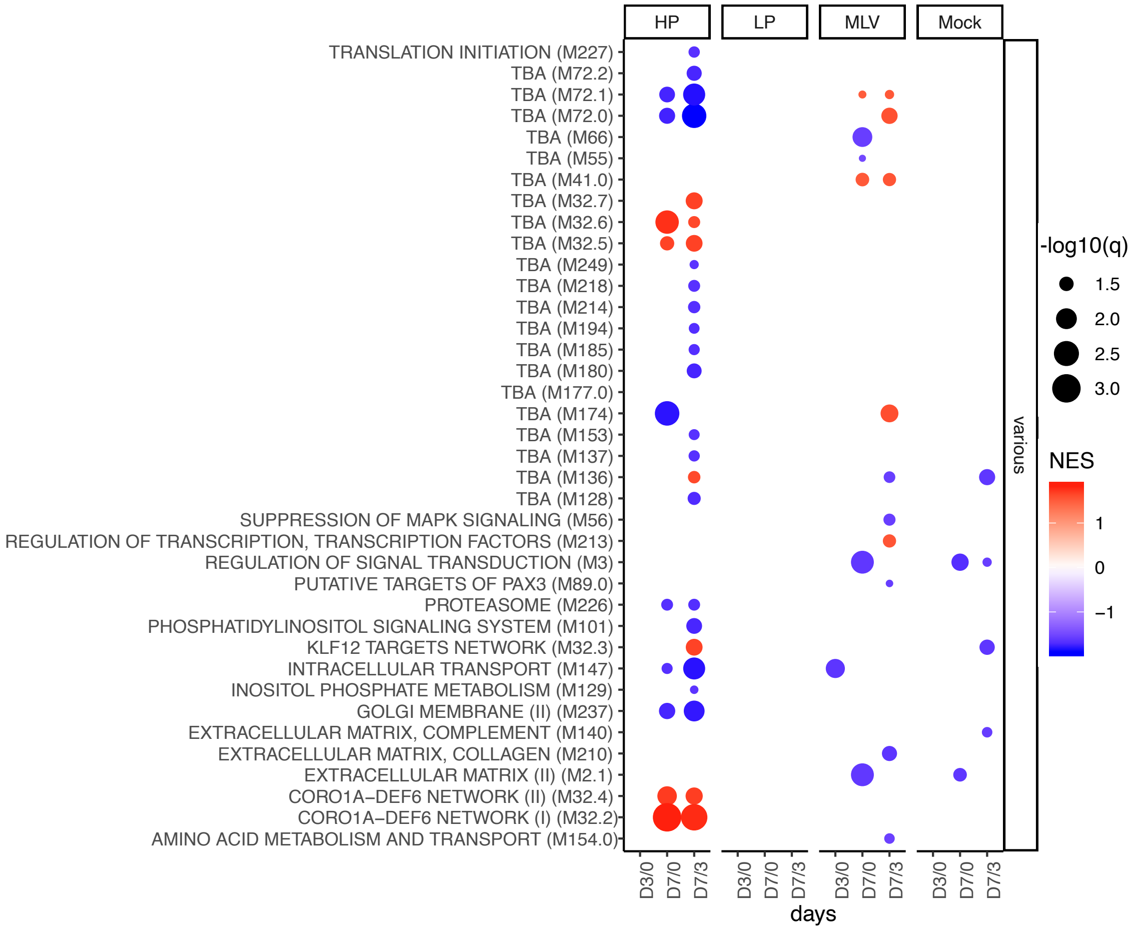

Supplementary Figure 3. Unclassified BTM perturbations induced by PRRSV and MLV. The bubble plot shows induction (red) or downregulation (blue) of BTM expression in PBMC following infection with HP, LP PRRSV2, MLV or MOCK injection. The changes in BTM activity were determined for D0 to D3 (D3/0), from D0 to D7 (D7/0) and from D3 to D7 (D7/3). Treatments are defined in the x-axis. A and B shows innate and adaptive BTM, respectively. On the left side of the heat map, the BTM families defined previously (Matthijs et al., 2019) are indicated. Circle sizes represent q-values and color intensity the normalized enrichment scores (NES). A cut-off of an FDR of q<0.05 was employed.

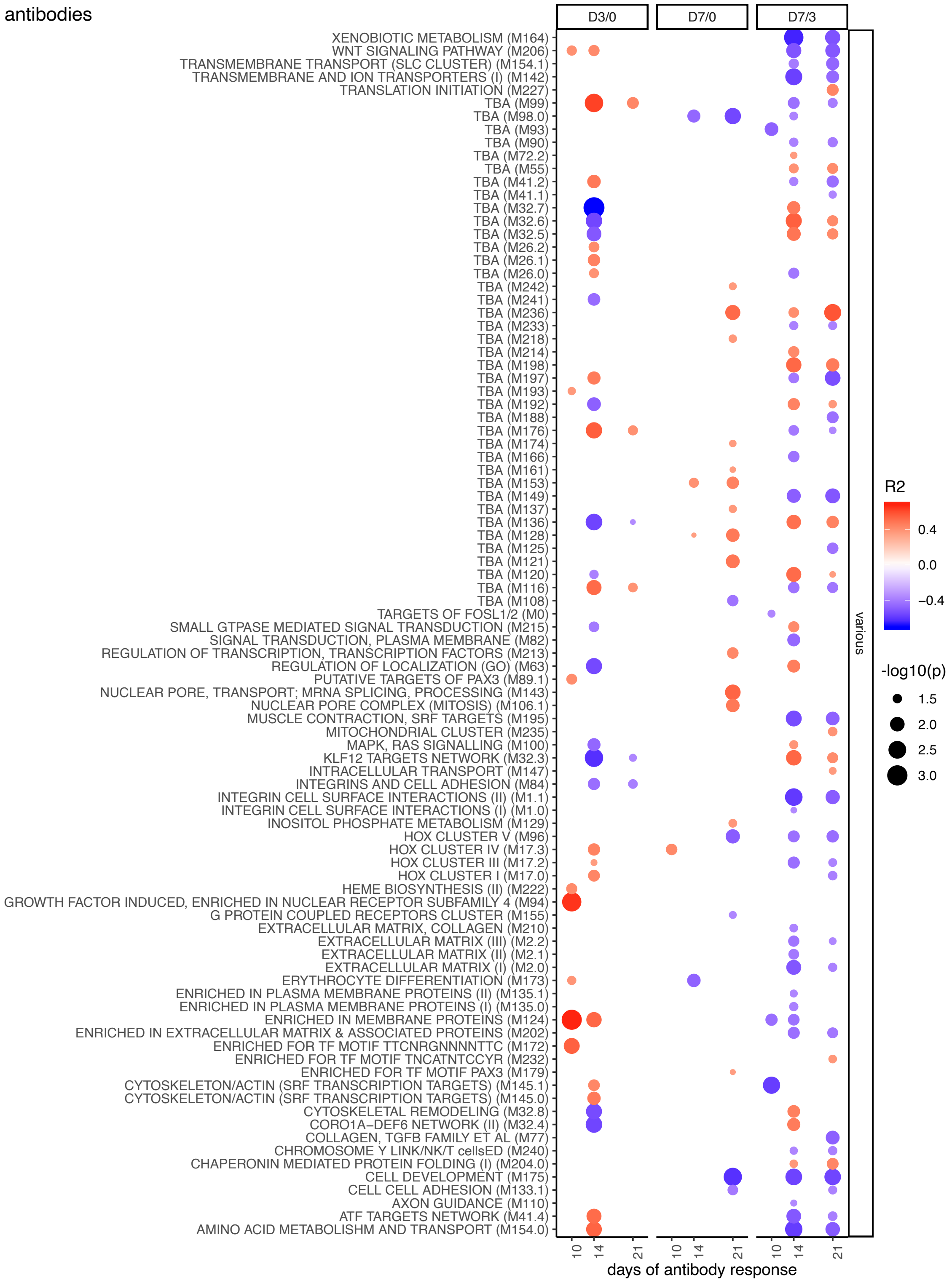

Supplementary Figure 4. Unclassified (“various”) BTM correlating with PRRSV inhibitory antibodies. Time-dependent single sample enrichment scores for BTM were correlated with virus inhibitory antibodies measured at 10, 14, and 21 days after LP or HP PRRSV-2 infection (x-axis, data from (Kick et al., 2019)). R squared correlation coefficients for the BTM changes from D3/0, D7/0 and D7/3 are indicated by the color gradient (red for positive values, blue for negative values). Increased circles sizes indicate smaller p values (cutoff < 0.05).

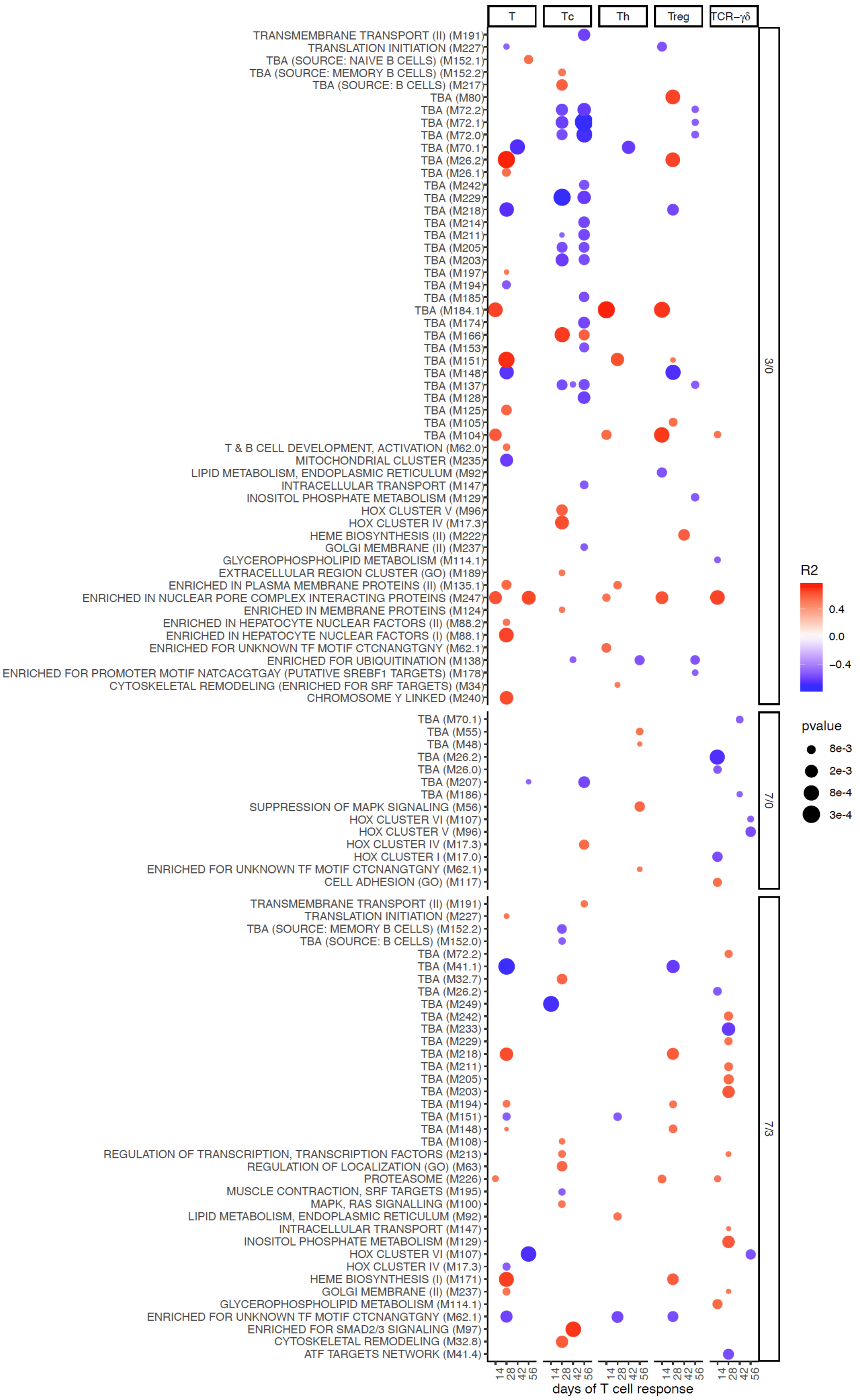

Supplementary Figure 5. Unclassified BTM correlating with T cell priming. Time-dependent single sample enrichment scores for BTM were correlated with the proliferative recall response of total CD3^{+} T cells, Tc, Th and TCR-γδ T-cells measured at 14, 28, 42 and 56 days after LP or HP PRRSV-2 infection (x-axis, data from (Kick et al., 2019)). R squared correlation coefficients for the BTM changes from D3/0, D7/0 and D7/3 are indicated by the color gradient (red for positive values, blue for negative values). Increased circles sizes indicate smaller p values (cutoff < 0.05). A shows IFN-I BTM and B inflammation BTM families (Matthijs et al., 2019).

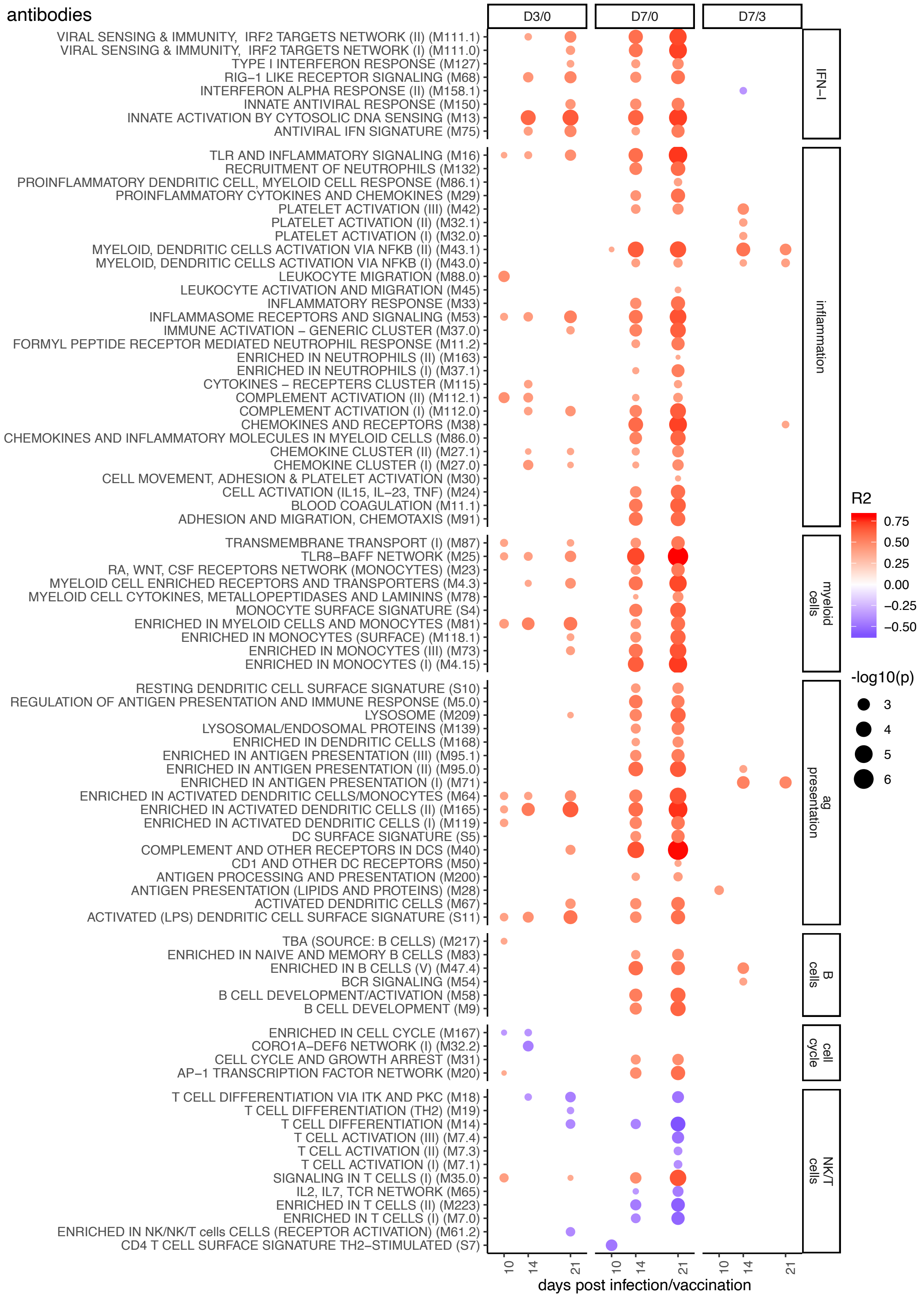

Supplementary Fig. 6. BTM correlating with anti-PRRSV inhibitory antibody levels. Time-dependent single sample enrichment scores for BTM were correlated with virus inhibitory antibodies measured at 10, 14, and 21 days after LP, HP PRRSV-2 infection or MLV vaccination (data pooled from all three groups (Kick et al., 2021)). R squared correlation coefficients for the BTM changes from D3/0, D7/0 and D7/3 are indicated by the color gradient (red for positive values, blue for negative values). Increased circles sizes indicate smaller p values (cutoff < 0.05). BTM were grouped in families (Matthijs et al., 2019).

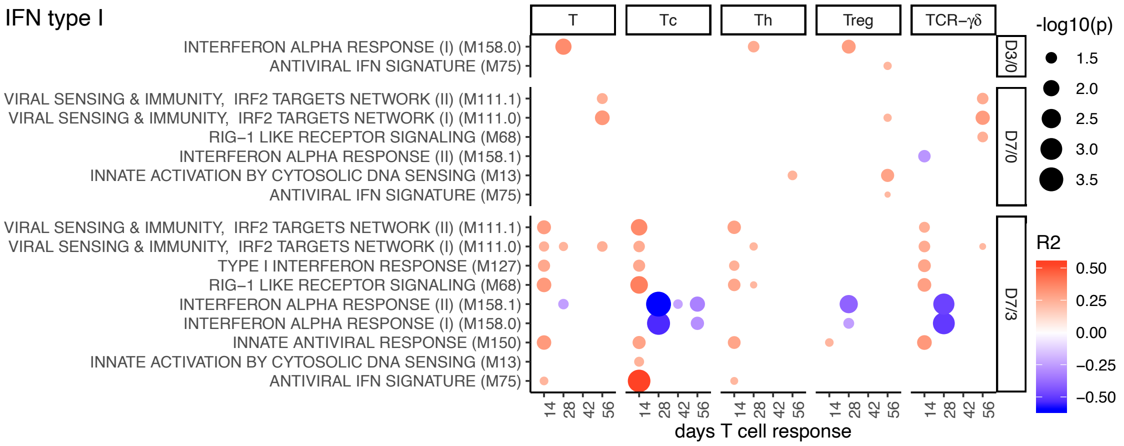

Supplementary Fig. 7. IFN-I BTM correlating with T cell priming. Time-dependent single sample enrichment scores for BTM were correlated with the proliferative recall response of total $CD3^+$ T cells, Tc, Th and TCR-γδ T-cells measured at 14, 28, 42 and 56 days after LP, HP PRRSV-2 infection or MLV vaccination (data pooled from all three groups, (Kick et. al 2019)). R squared correlation coefficients for the BTM changes from D3/0, D7/0 and D7/3 are indicated by the color gradient (red for positive values, blue for negative values). Increased circles sizes indicate smaller p values (cutoff < 0.05).

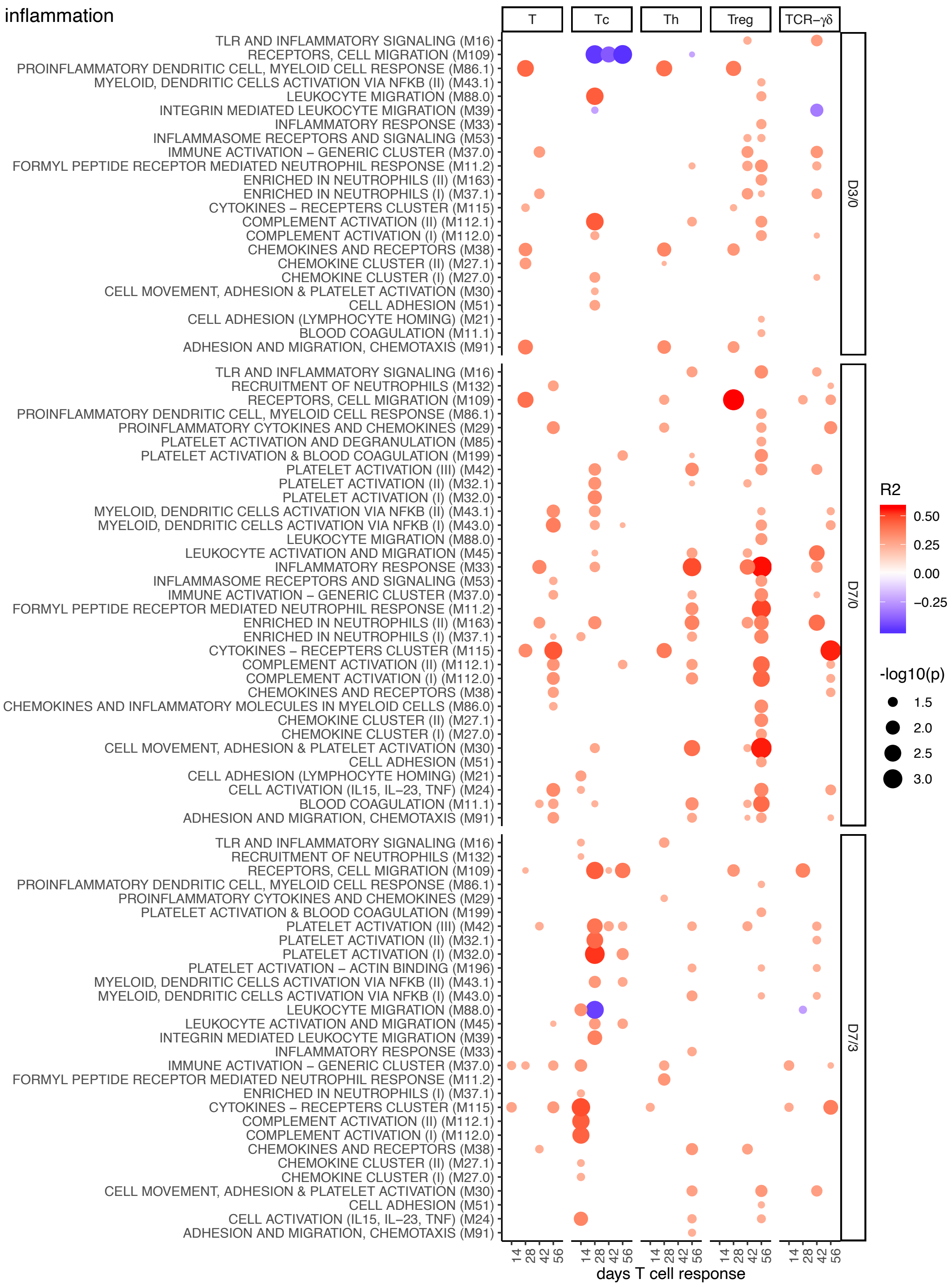

Supplementary Fig. 8. Inflammatory BTM correlating with T cell priming. Time-dependent single sample enrichment scores for BTM were correlated with the proliferative recall response of total CD3$^+$ T cells, Tc, Th and TCR-γδ T-cells measured at 14, 28, 42 and 56 days after LP, HP PRRSV-2 infection or MLV vaccination (data pooled from all three groups, (Kick et. al 2019)). R squared correlation coefficients for the BTM changes from D3/0, D7/0 and D7/3 are indicated by the color gradient (red for positive values, blue for negative values). Increased circles sizes indicate smaller p values (cutoff < 0.05).

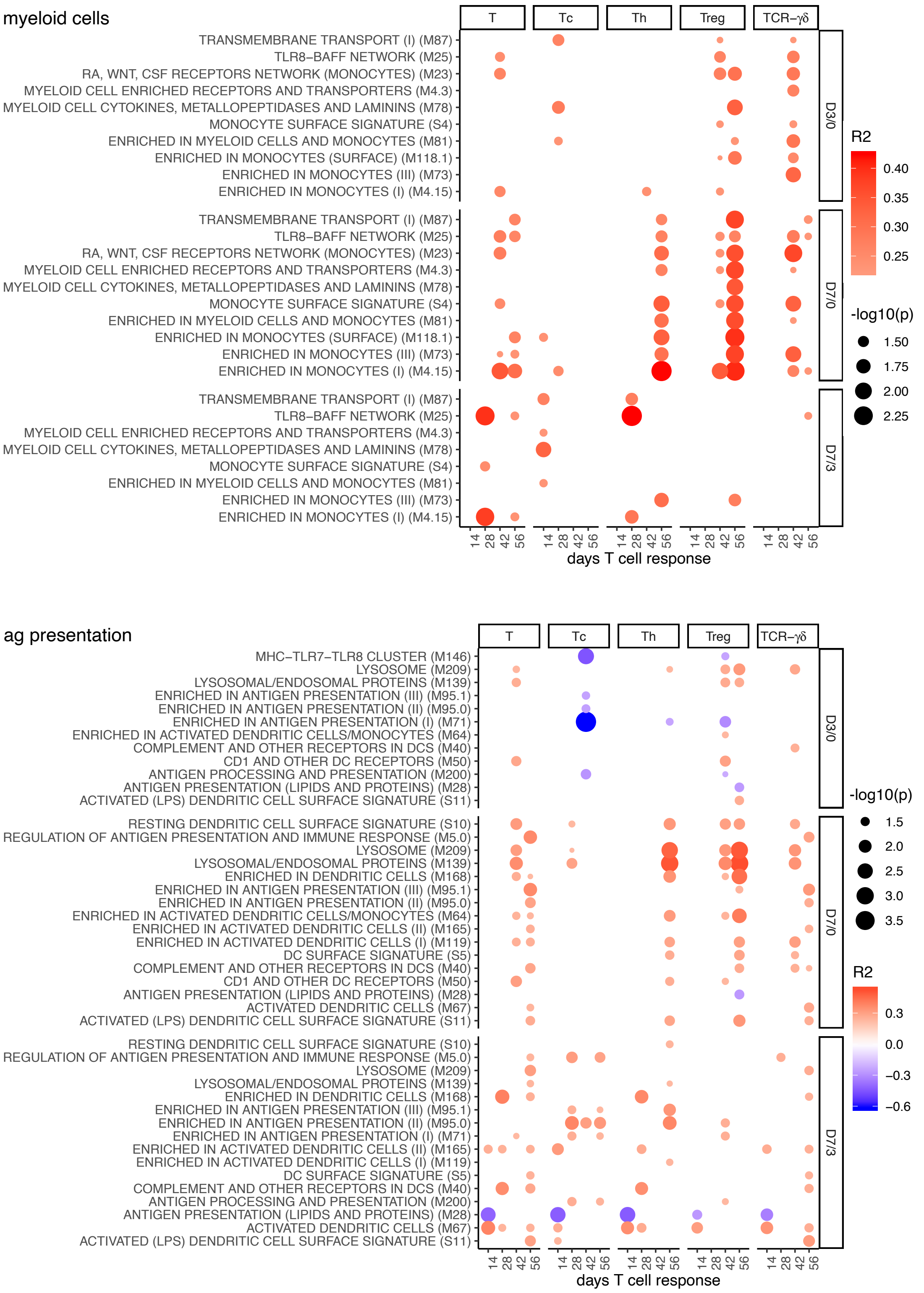

Supplementary Fig. 9. Myeloid cell and antigen presentation BTM correlating with T cell priming. Time-dependent single sample enrichment scores for BTM were correlated with the proliferative recall response of total $CD3^+$ T cells, Tc, Th and TCR-γδ T-cells measured at 14, 28, 42 and 56 days after LP, HP PRRSV-2 infection or MLV vaccination (data pooled from all three groups, (Kick et. al 2019)). R squared correlation coefficients for the BTM changes from D3/0, D7/0 and D7/3 are indicated by the color gradient (red for positive values, blue for negative values). Increased circles sizes indicate smaller p values (cutoff < 0.05).

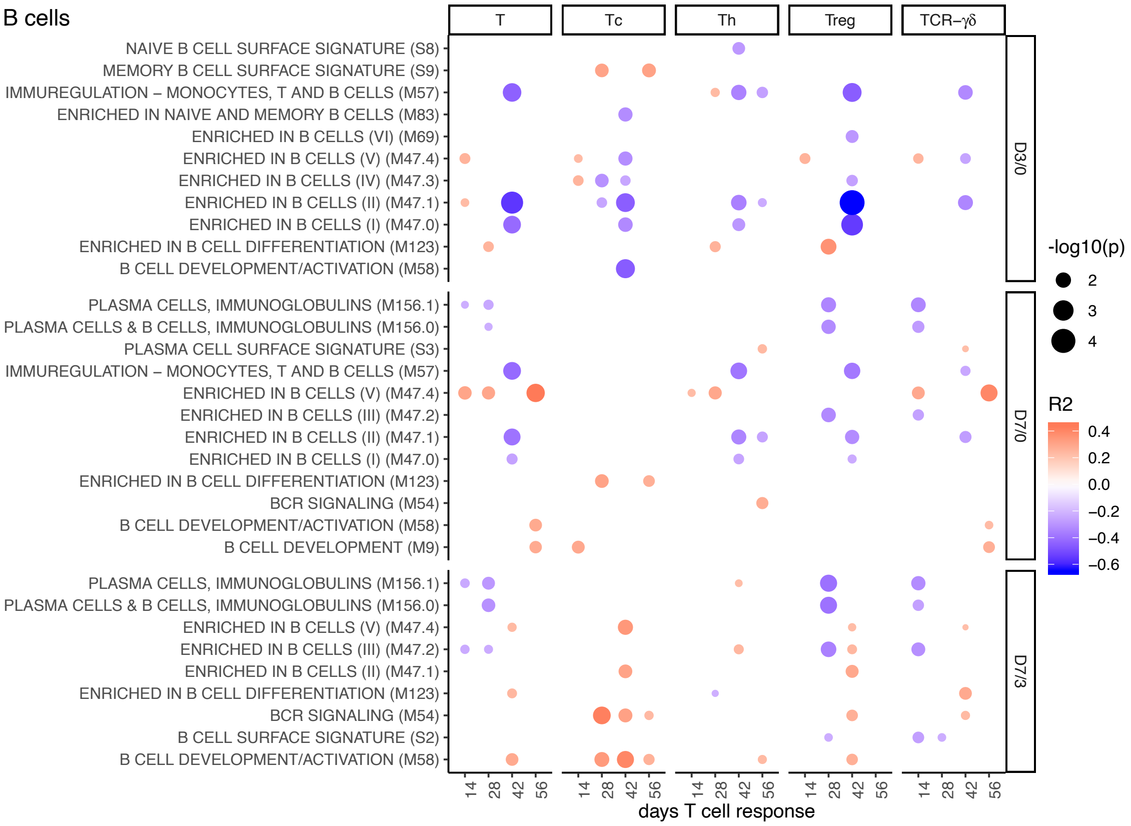

Supplementary Fig. 10. B cell BTM correlating with T cell priming. Time-dependent single sample enrichment scores for BTM were correlated with the proliferative recall response of total CD3$^+$ T cells, Tc, Th and TCR-γδ T-cells measured at 14, 28, 42 and 56 days after LP, HP PRRSV-2 infection or MLV vaccination (data pooled from all three groups, (Kick et. al 2019)). R squared correlation coefficients for the BTM changes from D3/0, D7/0 and D7/3 are indicated by the color gradient (red for positive values, blue for negative values). Increased circles sizes indicate smaller p values (cutoff < 0.05).

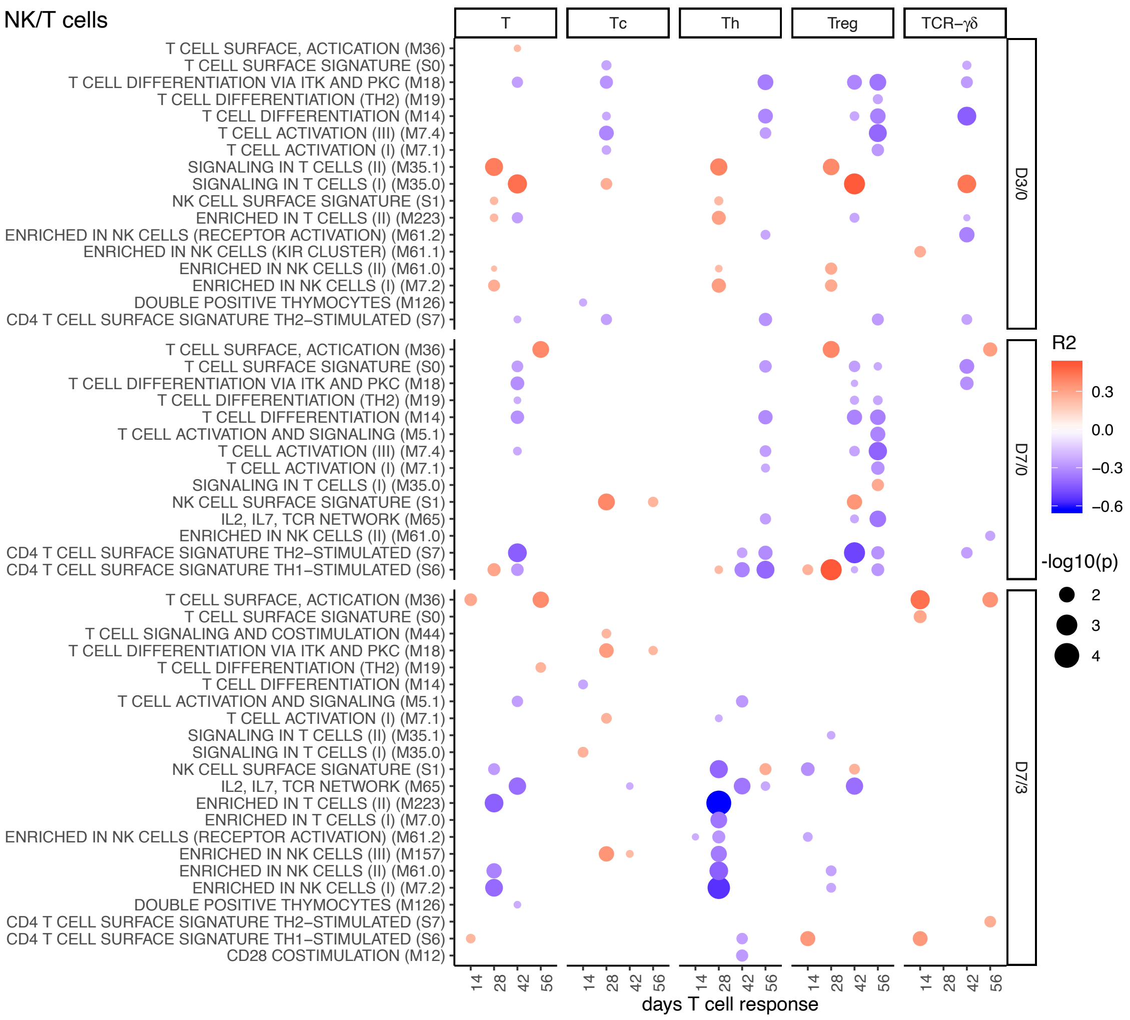

Supplementary Fig. 11. T/NK cell BTM correlating with T cell priming. Time-dependent single sample enrichment scores for BTM were correlated with the proliferative recall response of total $CD3^+$ T cells, Tc, Th and TCR-γδ T-cells measured at 14, 28, 42 and 56 days after LP, HP PRRSV-2 infection or MLV vaccination (data pooled from all three groups, (Kick et. al 2019)). R squared correlation coefficients for the BTM changes from D3/0, D7/0 and D7/3 are indicated by the color gradient (red for positive values, blue for negative values). Increased circles sizes indicate smaller p values (cutoff < 0.05).

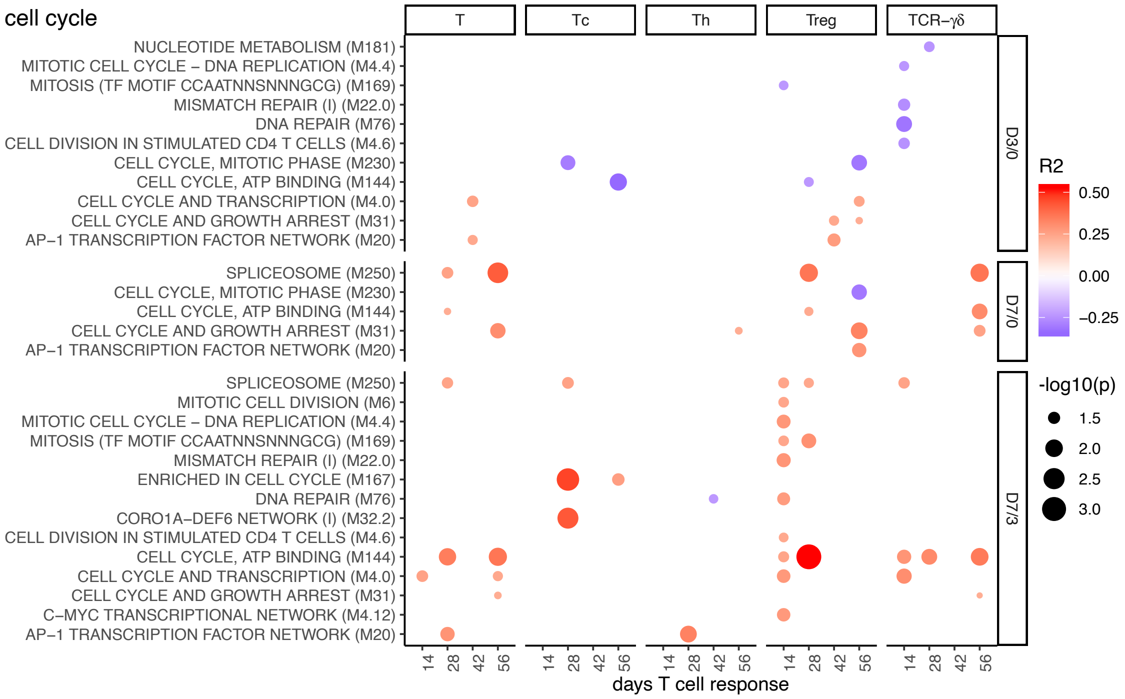

Supplementary Fig. 12. Cell cycle BTM correlating with T cell priming. Time-dependent single sample enrichment scores for BTM were correlated with the proliferative recall response of total CD3+ T cells, Tc, Th and TCR-γδ T-cells measured at 14, 28, 42 and 56 days after after LP, HP PRRSV-2 infection or MLV vaccination (data pooled from all three groups, (Kick et. al 2019)). R squared correlation coefficients for the BTM changes from D3/0, D7/0 and D7/3 are indicated by the color gradient (red for positive values, blue for negative values). Increased circles sizes indicate smaller p values (cutoff < 0.05).
